# Cell Lines in Myelodysplastic Syndromes/Neoplasms (MDS) Research: A Review of Existing Models and Their Applications

**DOI:** 10.3390/ijms27020898

**Published:** 2026-01-16

**Authors:** Karolina Maślińska-Gromadka, Małgorzata Palusińska, Julia Weronika Łuczak, Rafał Skopek, Leszek Kraj, Tino Schenk, Artur Zelent, Łukasz Szymański

**Affiliations:** 1 Department of Molecular Biology, Institute of Genetics and Animal Biotechnology, Polish Academy of Sciences, 05-552 Magdalenka, Poland; k.maslinska@igbzpan.pl (K.M.-G.); m.palusinska@igbzpan.pl (M.P.); j.luczak@igbzpan.pl (J.W.Ł.); leszek.kraj@wum.edu.pl (L.K.); a.zelent@igbzpan.pl (A.Z.); 2 Department of Human Physiology and Pathophysiology, Faculty of Medicine, Collegium Medicum, Cardinal Stefan Wyszynski University in Warsaw, 01-938 Warsaw, Poland; rafal.skopek@gmail.com; 3 Department of Oncology, Medical University of Warsaw, 02-091 Warsaw, Poland; 4 Department of Hematology/Oncology, Clinic of Internal Medicine II, Jena University Hospital, 07747 Jena, Germany; tino.schenk@med.uni-jena.de

**Keywords:** MDS cell lines, WHO, myelodysplastic syndromes, myelodysplastic neoplasms, CD markers, genetic rearrangements

## Abstract

Myelodysplastic syndromes/neoplasms (MDS) are clonal hematopoietic disorders characterized by ineffective hematopoiesis, cytopenias, and a variable risk of progression to secondary acute myeloid leukemia (sAML). Despite major advances in the molecular and clinical characterization of MDS, mechanistic and translational research remains constrained by the limited availability of well-validated in vitro models. Many historically used cell lines are difficult to maintain, exhibit restricted proliferative capacity, or represent advanced disease stages rather than bona fide MDS, while others have been affected by misidentification or cross-contamination. This review provides a comprehensive and critical overview of currently available MDS and MDS-related cell lines, including MDS92, MDS-L and its sublines, M-TAT, TER-3, SKK-1, SKM-1, and MOLM-17/18. We summarize their clinical origin, cytogenetic and molecular features, growth factor dependence, differentiation capacity, and experimental applications, with particular emphasis on their relevance to disease stage, clonal evolution, and leukemic transformation. In addition, we discuss the controversy surrounding misidentified models such as PC-MDS and highlight the importance of rigorous cell line authentication.

## 1. Introduction

### 1.1. Myelodysplastic Syndromes

Myelodysplastic syndromes/neoplasms (MDS) are a heterogeneous group of clonal hematopoietic disorders characterized by ineffective hematopoiesis, peripheral blood cytopenias, bone marrow dysplasia, and a variable risk of progression to secondary acute myeloid leukemia (sAML) [1,2]. These disorders result from genetic and epigenetic abnormalities that interfere with the normal development of blood cells and bone marrow function.

In 2022, the World Health Organization (WHO) updated the classification of hematologic malignancies, including MDS. As part of this revision, the terminology was refined to emphasize the neoplastic nature of these disorders, replacing the previous term “myelodysplastic syndromes” with “myelodysplastic neoplasms” (MDS) to align more closely with the nomenclature used for myeloproliferative neoplasms (MPN) [3]. Throughout this manuscript, we will refer to MDS as myelodysplastic syndromes to maintain consistency with the existing literature. MDS arises from hematopoietic stem and progenitor cells (HSPCs) that acquire somatic mutations conferring a clonal growth advantage and impairing normal differentiation. These malignant MDS stem cells (MDS-SC) initiate and drive disease progression [1]. Over time, additional mutations accumulate, leading to a progression from benign clonal hematopoiesis (CH) through clonal cytopenias (CCUS), eventually ending in MDS [2].

Genetic aberrations are central to MDS pathophysiology. Chromosomal abnormalities occur in 40–60% of cases and include deletions of chromosomes 5q and 7q, trisomy 8, and complex karyotypes [3]. Recurrent somatic mutations have been identified in more than 80% of patients, particularly in genes involved in RNA splicing: *SF3B1*, *SRSF2*, *U2AF1*, *ZRSR2*; epigenetic regulation: *TET2*, *DNMT3A*, *IDH1/2*, *EZH2*, *ASXL*; signal transduction: *JAK2*; and tumor suppression: *TP53* (associated with poor prognosis) [4].

These genetic alterations contribute to deregulated hematopoiesis, increased cell apoptosis, and leukemic transformation. One of the significant mutations in MDS involves the *ASXL1* gene. Mutations in exon 12 of *ASXL1* may act through gain-of-function mechanisms and are associated with a worse prognosis for patients [3].

Diagnosis of MDS is often difficult because of overlapping clinical and genetic features shared with other myeloid malignancies. In particular, MDS can resemble or transform into disorders such as aplastic anemia (AA), especially in cases with bone marrow hypocellularity and pancytopenia, MPNs, and sAML. MDS with excess blasts can evolve into AML, often preceded by the appearance of leukemic clones months before clinical transformation [5].

Genetic tools such as next-generation sequencing (NGS) are becoming increasingly important in distinguishing MDS from other hematopoietic disorders [5].

MDS is predominantly a disease of older adults, with a median age at diagnosis of over 70 years. Incidence increases with age and is estimated at approximately 4 per 100,000 persons annually in the United States [2]. The clinical presentation varies widely, from asymptomatic cytopenias to severe pancytopenia and transfusion dependency.

Disease risk and prognosis are determined using systems like the Revised International Prognostic Scoring System (IPSS-R), incorporating cytogenetics, blast count determination, and degree of cytopenia. These tools help guide treatment decisions, including adjuvant therapy, administration of hypomethylating drugs, or allogeneic hematopoietic stem cell transplantation in eligible patients.

Although understanding of MDS stem cell biology has increased, translation into effective, targeted therapies remains limited [1]. Current strategies include supportive treatment consisting of red blood cell and platelet transfusions and administration of growth factors, disease-modifying therapies such as hypomethylating agents (e.g., azacitidine and decitabine) and lenalidomide, especially in del(5q) MDS, and targeted therapy with luspatercept for anemia in MDS-RS. Efforts to use genetic testing in treatment selection may enable more individualized and effective health care.

Despite substantial advances in the molecular and clinical characterization of myelodysplastic syndromes, studying MDS remains particularly challenging due to pronounced biological heterogeneity, age-related disease onset, clonal evolution, and the strong influence of the bone marrow microenvironment. An additional and critical limitation is the scarcity of well-characterized and disease-representative MDS cell lines. Many available in vitro models are difficult to establish and maintain, show limited proliferative capacity, or represent advanced stages of disease, including secondary AML, rather than bona fide MDS. In particular, cell lines such as SKM-1 and MOLM-17 and MOLM-18, which are frequently used in MDS-oriented studies, were derived after leukemic transformation and therefore primarily reflect biological processes associated with clonal evolution and disease progression rather than the ineffective hematopoiesis that defines primary MDS, especially lower-risk subtypes. Furthermore, controversies persist regarding the cellular origin of existing MDS cell lines and the extent to which they faithfully recapitulate primary disease biology. The aim of this manuscript is to critically evaluate the current data on MDS cell line models and to provide a comprehensive overview of the key characteristics, origin, and applications of existing MDS-derived cell lines, thereby facilitating their appropriate use and interpretation in preclinical research.

### 1.2. Differences Between MDS and AML

MDS is generally defined by ineffective hematopoiesis and various degrees of cytopenias resulting from defective differentiation and maturation of hematopoietic progenitors [6,7]. In contrast, AML is characterized by a marked accumulation of immature myeloid blasts in the bone marrow and peripheral blood, representing an aggressive proliferation of malignant clones [8,9,10]. The percentage of blasts is a key diagnostic criterion distinguishing the two diseases: in MDS, the blast count remains below 20%, whereas a count exceeding this threshold defines AML [8,10,11]. As stated above, MDS can evolve into AML, particularly in higher-risk cases, with a progressive increase in blast percentage and worsening clinical course [8,11,12].

At the molecular level, MDS is associated with mutations in genes involved in chromatin remodeling and the spliceosome machinery, which contribute to inefficient hematopoiesis and multilineage dysplasia [9,10,11]. AML, on the other hand, often results from mutations in signaling pathway genes, such as FLT3 or RAS, which are associated with rapid clonal expansion and leukemic transformation, both de novo and secondary to prior MDS [9,10,11]. Clinically, patients with MDS often present with symptoms associated with cytopenias, including fatigue, recurrent infections, and a tendency to bleed. The prognosis of MDS is difficult to determine, as the disease can take on a mild form with prolonged survival or an aggressive subtype with rapid transformation to AML [7,13,14]. Patients with AML usually have more severe systemic symptoms such as fever, fatigue, and hemorrhagic complications due to the high leukemic burden [9,13,15], and the overall prognosis is worse compared to MDS.

Treatment strategies also differ widely between the two conditions. In MDS, therapeutic decisions are risk-adjusted; lower-risk patients are often treated with supportive care, including transfusions and growth factors, while higher-risk patients may require disease-modifying drugs or allogeneic stem cell transplantation to delay progression [14,16]. On the other hand, AML requires rapid and intensive therapy, usually consisting of induction chemotherapy followed by consolidation with additional chemotherapy or hematopoietic stem cell transplantation [8,15]. While MDS may remain stable for a longer time in some patients, the risk of progression to AML, particularly in those with unfavorable cytogenetics or high blast counts, underscores the importance of early risk stratification and appropriate therapeutic intervention [9,12].

Importantly, these fundamental biological differences between MDS and AML have direct implications for experimental modeling. While AML-derived cell lines are characterized by autonomous proliferation and dominant leukemic clones, MDS, particularly lower-risk disease, is defined by ineffective hematopoiesis, increased apoptosis, and a strong dependence on extrinsic microenvironmental cues. Consequently, findings obtained using AML cell lines cannot be directly extrapolated to MDS biology without careful consideration. Although AML-based models can be informative for investigating clonal evolution, leukemic transformation, and therapeutic resistance during progression from MDS to secondary AML, they do not substitute for bona fide MDS cell lines when studying primary disease mechanisms, especially those relevant to low-risk MDS.

### 1.3. Different Types of MDS

Over the years, the classification of MDS has undergone significant refinement, beginning with the French-American-British (FAB) system and later enhanced by successive iterations of the World Health Organization criteria [17].

The 2022 revision of the WHO classification of MDS provides a comprehensive framework that integrates both morphological and genetic features, allowing for more precise diagnosis and prognostic stratification. MDS with low blast count (MDS-LB) is defined by less than 5% myeloblasts in the bone marrow and less than 2% in peripheral blood. This subtype generally presents with cytopenias and an indolent clinical course, although disease progression remains possible. MDS with increased blasts (MDS-IB) includes cases with 5–9% myeloblasts in the bone marrow (2–4% in peripheral blood; MDS-IB1) or 10–19% in the bone marrow (5–19% in peripheral blood; MDS-IB2). Patients in this category are at higher risk of progression to AML and typically exhibit a more aggressive clinical course.

Genetically defined MDS subtypes highlight the prognostic significance of recurrent molecular abnormalities. MDS-5q is associated with an isolated deletion of the long arm of chromosome 5, often presenting with macrocytic anemia and a favorable response to targeted therapies. MDS-SF3B1 is characterized by mutations in SF3B1 and frequent ring sideroblasts, generally corresponding to a favorable prognosis. MDS-bi TP53 results from bi-allelic TP53 inactivation, often accompanied by complex karyotypes and poor overall survival [18].

Hypoplastic MDS (MDS-h) is distinguished by markedly reduced bone marrow cellularity (<30%) and may resemble aplastic anemia, with unique molecular and immunologic characteristics compared to other MDS subtypes. This updated classification improves risk stratification, enhances prognostic accuracy, and supports individualized management and therapeutic decision-making for patients with MDS [18].

For atypical presentations that do not conform to established categories, MDS, unclassifiable (MDS-U), serves as a diagnostic category, encompassing rare morphologic or cytogenetic findings. Additionally, therapy-related MDS (t-MDS) is acknowledged as a distinct clinical entity due to its etiologic link with prior cytotoxic chemotherapy or radiation exposure [17]. Overlapping syndromes, such as mixed myelodysplastic/myeloproliferative neoplasms (MDS/MPN), including chronic myelomonocytic leukemia (CMML), exhibit dual features of dysplasia and proliferation and are considered within the broader diagnostic spectrum [17]. The WHO classification system thus plays a central role in the diagnostic workup, prognostic stratification, and therapeutic planning for MDS patients, offering a structured and biologically informed approach to understanding the diverse clinical manifestations of the disease [19].

## 2. MDS Cell Lines

**Figure 1:**
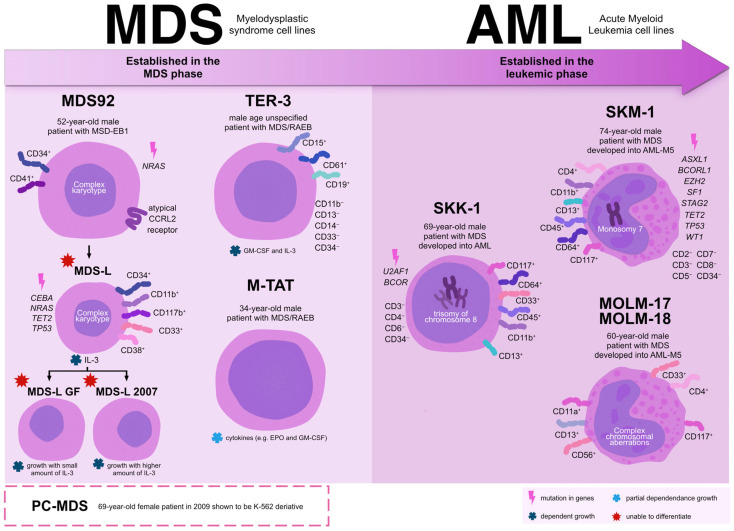
Disease stage and clonal progression represented by MDS and MDS-related cell line models. The schematic positions myelodysplastic syndrome and MDS-related cell lines along the disease continuum from primary MDS to secondary acute myeloid leukemia according to their stage of establishment. Cell lines on the left were established during the MDS phase and retain features of dysplastic and ineffective hematopoiesis, including cytokine dependence and limited differentiation capacity, whereas cell lines on the right were established after leukemic transformation and primarily represent secondary AML with increased proliferative capacity and complex genetic abnormalities. The left-to-right gradient illustrates clonal evolution and progression toward AML, highlighting increasing loss of differentiation and growth factor independence. Pink lightning symbols indicate reported gene mutations, blue symbols denote partial or complete growth factor dependence, and red star symbols indicate impaired differentiation capacity.

Research in the field of MDS poses significant challenges, particularly due to the limited availability of well-characterized MDS cell lines. Accurate and rigorous characterization of these models is essential to ensure that they faithfully represent the biological and genetic features of the disease, which requires comprehensive cytogenetic and molecular genetic analyses [20,21]. Despite these limitations, several cell lines derived from patients with MDS or MDS-associated disease progression have been established and provide valuable experimental systems for investigating disease pathogenesis and evaluating potential therapeutic strategies. It should be emphasized that currently available in vitro models span a broad biological spectrum, ranging from bona fide MDS-derived cell lines to models established after leukemic transformation. While the former retain key features of dysplastic and ineffective hematopoiesis, the latter predominantly reflect advanced disease stages characterized by increased blast proliferation and partial or complete loss of MDS-defining phenotypes. Accordingly, AML-derived or secondary AML cell lines included in this review should be regarded primarily as models of MDS progression rather than as faithful representations of primary MDS, particularly low-risk disease. A defining technical characteristic of most bona fide MDS-derived cell lines is their strong dependence on exogenous cytokines, including interleukin-3, granulocyte-macrophage colony-stimulating factor, or erythropoietin, for survival and proliferation. While this requirement reflects the biological reliance of dysplastic hematopoietic cells on microenvironmental support, it also imposes important experimental constraints. Cytokine supplementation complicates assay standardization, increases variability between laboratories, and limits the feasibility of large-scale or high-throughput experimental approaches. In addition, many MDS cell lines display slow proliferation kinetics and limited long-term stability. For example, MDS92 exhibits a population doubling time of approximately 80 to 90 h, which restricts experimental throughput and increases resource demands. Phenotypic instability during extended culture further complicates reproducibility, particularly in studies requiring prolonged drug exposure or serial sampling. As a consequence, bona fide MDS cell lines are best suited for mechanistic investigations focusing on differentiation defects, epigenetic dysregulation, and cytokine-dependent signaling pathways rather than large-scale pharmacological screening. Key clinical, cytogenetic, molecular, and phenotypic characteristics of the MDS and MDS-related cell lines discussed in this review are summarized in Figure 1.

To facilitate direct comparison across available models and to support informed model selection, a comprehensive overview of clinical origin, molecular features, growth requirements, authentication status, and validated experimental applications of all discussed cell lines is provided in Table 1.

### 2.1. MDS92

MDS92 is a myeloid cell line that originated from the bone marrow of a patient before leukemic transformation. It was originally derived from the bone marrow of a 52-year-old male patient diagnosed with MDS-EB1 (MDS with excess of blasts type 1) [32]. MDS92 cells are composed of blastic cells and dysplastic myeloid cells and have been shown to contain multiple hematopoietic lineage populations, including cells with neutrophil-like and macrophage-like characteristics, reflecting multilineage differentiation potential typical of MDS. In culture, cell growth and lineage composition are generally maintained over time, and cells preserve unique molecular and cytogenetic characteristics. MDS92 is a growth-factor–dependent cell line, requiring interleukin-3 (IL-3) for sustained proliferation, and exhibits slow growth kinetics, with a reported population doubling time of approximately 80–90 h, consistent with its preleukemic phenotype.

The MDS92 cell line exhibits complex karyotypic abnormalities, including deletions involving chromosomes 5 and 7, together with a point mutation at codon 12 of the NRAS oncogene. In addition to NRAS activation, molecular profiling data have reported alterations in genes recurrently implicated in high-risk MDS, including TP53 and CEBPA, further supporting the relevance of MDS92 as a genetically complex preleukemic model [Cellosaurus, MDS92 cell line]. These genetic features are characteristic of a preleukemic state and are consistent with the biological properties of advanced MDS. MDS92 is utilized to study mechanisms of resistance to DNA methyltransferase (DNMT) inhibitors, such as azacitidine, which are commonly used in the treatment of high-risk MDS [33]. Research has shown that the atypical chemokine receptor CCRL2 is upregulated in MDS92 cells. CCRL2 modulates epigenetic regulatory pathways, including DNMT-associated programs, and influences the sensitivity of MDS92 cells to azacitidine [23].

### 2.2. MDS-L

MDS-L represents a blastic subclone derived from the MDS92 cell line and constitutes a unique experimental model of advanced MDS. A recent integrated cytogenetic and genomic analysis confirmed that MDS-L is the only currently available cell line definitively originating during the MDS phase rather than after leukemic transformation, identifying two related clonal populations characterized by highly complex karyotypes and recurrent mutations in CEBPA, NRAS, TET2, and TP53 [26].

MDS-L is widely used to investigate MDS biology and to evaluate candidate therapeutic agents. The cell line proliferates in the presence of IL-3 but, in contrast to its parental MDS92 line, has lost its differentiation capacity, consistent with a more advanced, blast-dominated disease stage [24,26]. Morphologically, MDS-L cells display features typical of myeloid blasts, including a high nuclear-to-cytoplasmic ratio and immature chromatin structure [34].

MDS-L has been extensively employed in preclinical drug development studies. Several reports have demonstrated that withaferin A (WFA) and rigosertib induce apoptosis in MDS-L cells through multiple mechanisms, including reactive oxygen species (ROS) generation, disruption of survival signaling pathways, and induction of mitotic stress, supporting the utility of this model for evaluating targeted and stress-based therapies [32,35].

Two sublines of MDS-L have been described: MDS-LGF and MDS-L-2007. These sublines differ markedly in their cytokine requirements. MDS-LGF cells are capable of sustained proliferation with minimal IL-3 supplementation (~1 ng/mL), whereas MDS-L-2007 cells require substantially higher IL-3 concentrations (~100 ng/mL) for growth. Functional studies indicate that MDS-LGF cells represent an effective in vitro model of an indolent but high-risk MDS phenotype, characterized by blast expansion with partial cytokine independence [36].

Overall, the MDS-L cell line constitutes a valuable system for studying MDS pathophysiology, clonal evolution, and therapeutic vulnerability. Its blastic phenotype, genetic complexity, and graded cytokine dependence across sublines make MDS-L particularly well suited for both in vitro and in vivo preclinical studies aimed at developing and validating targeted therapies for high-risk MDS.

### 2.3. M-TAT

The M-TAT cell line was established from the peripheral blood of a 3-year-old pediatric patient diagnosed with MDS presenting as refractory anemia with excess blasts (RAEB). M-TAT is a cytokine-dependent cell line, requiring growth factors such as erythropoietin (EPO) and granulocyte–macrophage colony-stimulating factor (GM-CSF) for sustained proliferation and survival. Under appropriate cytokine stimulation, M-TAT cells retain the capacity to undergo partial differentiation along erythroid and megakaryocytic lineages, reflecting key functional features of dysplastic hematopoiesis [37].

In the classification proposed by Drexler et al., M-TAT is included among the so-called “valid MDS cell lines”, a limited group of models considered representative of the MDS phenotype. This group also includes TER-3, MDS92, and their respective derivatives [22]. This designation underscores the relevance of M-TAT as a bona fide MDS model rather than a secondary leukemia-derived line.

In preclinical research, the M-TAT cell line serves as a valuable biological system for studying the role of microenvironmental growth signals in the regulation of proliferation and differentiation of dysplastic hematopoietic cells. Its pronounced responsiveness to cytokine stimulation makes it particularly useful for dissecting growth factor–dependent signaling pathways and for evaluating therapeutic strategies aimed at modulating ineffective hematopoiesis in MDS.

Nevertheless, M-TAT shares limitations common to MDS-derived cell lines. Its strict dependence on exogenous cytokines constrains long-term culture conditions, and its genetic and functional profile cannot fully capture the molecular and clinical heterogeneity observed across MDS patient populations. Despite these limitations, M-TAT remains a relevant and informative in vitro model for investigating MDS pathogenesis and for testing novel therapeutic approaches targeting dysregulated growth and differentiation.

### 2.4. TER-3

The TER-3 cell line is a hematopoietic model established from a patient with MDS refractory to therapy, presenting with refractory anemia with excess blasts (RAEB). TER-3 cells are cytokine dependent, requiring GM-CSF and IL-3 for survival and proliferation, underscoring their reliance on extrinsic growth signals characteristic of dysplastic hematopoiesis.

Cytochemical analyses demonstrated weak positivity for dianisidine and nonspecific esterase, with no detectable peroxidase activity, consistent with an immature hematopoietic phenotype. Immunophenotypic profiling revealed strong expression of lineage-associated markers spanning multiple hematopoietic programs, including CD15 (myeloid), CD19 (lymphoid), and CD61 (megakaryocytic). In contrast, commonly expressed myeloid progenitor and stem cell markers such as CD13, CD33, and CD34 were absent. This unusual antigenic pattern supports a multilineage differentiation phenotype and suggests leukemic transformation at the level of a multipotent hematopoietic progenitor, a feature relevant to advanced-stage MDS biology.

Functional differentiation studies further demonstrated that exposure to granulocyte colony-stimulating factor (G-CSF), but not IL-3, increased the proportion of dianisidine-positive and nonspecific esterase–positive cells, indicating selective responsiveness to G-CSF–mediated differentiation cues. These properties highlight the utility of the TER-3 cell line as a model system for investigating G-CSF–driven hematopoietic differentiation and activation mechanisms in the context of dysplastic and preleukemic hematopoiesis [27].

### 2.5. SKK-1

The SKK-1 line was established from cancer cells obtained from a patient with progressive MDS that subsequently evolved into acute leukemia [28]. Cytogenetic analysis revealed a numerical gain of chromosome 8 (trisomy 8) as a major abnormality, together with additional structural chromosomal alterations. The reported karyotype is 47,XY,add(4)(p16),+8,add(12)(p13), consistent with genomic instability associated with advanced MDS and leukemic transformation [38].

Immunophenotypic characterization demonstrated that SKK-1 cells express canonical myeloid markers commonly used in the diagnosis of myeloid neoplasms. The cells are positive for the progenitor-associated marker CD117 (KIT) and the monocyte-lineage marker CD64, while lacking markers of terminal differentiation, indicating an immature myeloid phenotype [38]. This immunoprofile supports the use of SKK-1 as a model for evaluating therapeutic strategies aimed at inducing monocytic differentiation, which can be monitored by the acquisition of late monocytic markers such as CD14 [28].

High-resolution genomic profiling using single-nucleotide polymorphism (SNP) microarrays and targeted sequencing of an 83-gene MDS/AML-associated panel identified recurrent somatic alterations in SKK-1. Notably, a mutation in the U2AF1 splicing factor gene was detected (c.101C>T; p.Ser34Phe), a hotspot mutation frequently observed in MDS and secondary AML and associated with aberrant RNA splicing and disease progression [38].

Collectively, SKK-1 represents a valuable experimental model for investigating the molecular and cellular mechanisms underlying MDS progression to acute myeloid leukemia. Its combination of stable cytogenetic abnormalities, particularly trisomy 8, together with functionally relevant mutations in RNA splicing machinery and a well-defined immature myeloid immunophenotype, makes SKK-1 well suited for studies of leukemic transformation and therapeutic intervention.

### 2.6. SKM-1

This cell line is derived from a patient with MDS that progressed to monoblastic leukemia and therefore represents a model of leukemic transformation arising in the context of antecedent MDS rather than primary disease [28,39,40,41]. Since its initial description, SKM-1 has been widely used in experimental studies addressing disease progression, molecular alterations, and therapeutic response in high-risk MDS and secondary acute myeloid leukemia. The clinical origin of SKM-1 places it biologically closer to secondary AML, which is reflected in its growth characteristics, genetic complexity, and loss of features associated with ineffective hematopoiesis.

At the molecular level, SKM-1 harbors recurrent mutations in genes frequently implicated in MDS pathogenesis and disease progression, including *TET2*, *ASXL1*, and *TP53*, which are commonly associated with high-risk disease and adverse prognosis [39]. These alterations support the relevance of SKM-1 as a genetically representative model of advanced myeloid malignancy evolving from MDS. Cytogenetic analyses have demonstrated a complex karyotype, consistent with genomic instability characteristic of leukemic transformation rather than early-stage MDS [28,41]. From an experimental perspective, SKM-1 displays robust proliferative capacity and does not require continuous cytokine supplementation for in vitro propagation, distinguishing it from bona fide MDS-derived cell lines such as MDS92 or MDS-L that retain strong dependence on exogenous growth factors. These properties have facilitated the widespread use of SKM-1 in functional assays, signaling pathway analyses, and pharmacological testing. In particular, SKM-1 has been employed extensively to investigate mechanisms of resistance to hypomethylating agents, including azacitidine, through the development and characterization of drug-sensitive and drug-resistant sublines [39].

SKM-1 has also served as a versatile platform for mechanistic studies examining apoptosis, autophagy, and intracellular signaling pathways relevant to myeloid malignancies. Gene silencing and pathway modulation approaches in SKM-1 cells have provided insights into the functional consequences of specific genetic alterations and have contributed to the identification of candidate therapeutic targets [42,43]. In addition, differentiation-inducing compounds have been evaluated in this model, further supporting its utility for studying leukemic cell plasticity in advanced disease contexts [44].

Taken together, SKM-1 should be regarded primarily as a model of MDS-to-AML progression and secondary AML biology rather than as a faithful in vitro representation of bona fide MDS, particularly lower-risk subtypes. Its major strengths include experimental scalability, genetic relevance to high-risk disease, and suitability for signaling-focused and pharmacological studies. Its principal limitation lies in the absence of key features of primary MDS, including ineffective hematopoiesis and strict cytokine dependence, which must be considered when interpreting findings derived from this model.

### 2.7. PC-MDS

The PC-MDS cell line was initially reported as the first human cell line derived from the bone marrow of a patient with myelodysplastic syndrome, specifically therapy-related MDS (t-MDS), who did not develop overt leukemia after the MDS phase [20,45]. However, the biological origin of PC-MDS has since been the subject of significant controversy. A comprehensive re-evaluation published in 2009 demonstrated that PC-MDS was contaminated and is in fact derived from the K562 cell line, an erythroleukemia model of acute myeloid leukemia, rather than from primary MDS cells [22].

Despite its initial characterization as an MDS-derived model, PC-MDS consists of rapidly proliferating mononuclear cells displaying features consistent with early myeloid precursor cells. The reported immunophenotype includes expression of CD13, CD15, CD30, CD33, and CD45, markers frequently associated with myeloid lineage cells and originally interpreted as supportive of an MDS origin [20]. Cytogenetic analyses described numerical and structural chromosomal abnormalities, including alterations involving chromosomes 5, 7, and 20, as well as a biallelic hypermethylation pattern of the MGMT promoter, which was used to investigate epigenetic dysregulation in MDS [45].

In light of the confirmed K562 origin, findings generated using PC-MDS must be interpreted with caution when extrapolated to primary MDS biology. While the line may still be useful for studying general myeloid leukemia biology, epigenetic regulation, and drug response mechanisms, it should not be considered a valid MDS cell line. Its inclusion in contemporary MDS-focused studies requires explicit acknowledgment of its true identity and limitations [46]. In summary, although PC-MDS was historically described as a key MDS model, current evidence indicates that it represents a misidentified leukemia-derived cell line. This case underscores the importance of rigorous cell line authentication in preclinical research and highlights the need to rely on well-validated MDS models when investigating disease-specific mechanisms and therapeutic responses [47].

## 3. Alternative Models Used in MDS Research

The availability of bona fide MDS cell lines remains limited, and their use is further constrained by substantial technical challenges associated with in vitro propagation, including low proliferative capacity, cytokine dependence, and phenotypic instability during prolonged culture. These limitations restrict experimental scalability and complicate both mechanistic investigations and drug-response studies. In addition, persistent concerns regarding cell line authentication continue to affect the field, as misidentification, cross-contamination, and cumulative genetic and epigenetic drift during long-term culture have been documented for several historically used MDS models, thereby undermining reproducibility and complicating interpretation of published data. These challenges highlight the need for rigorous validation and cautious interpretation when employing MDS-derived cell lines. As a consequence of these limitations, many investigators extend their experimental frameworks to include AML-derived cell lines, particularly those originating from secondary AML or exhibiting molecular, cytogenetic, or phenotypic features overlapping with high-risk MDS. While such models do not recapitulate the ineffective hematopoiesis characteristic of primary MDS, they provide practical and biologically informative systems for studying clonal evolution, leukemic transformation, and therapeutic response in advanced stages of disease. Their use therefore requires explicit recognition that they model MDS-to-AML progression rather than bona fide MDS biology.

### 3.1. MOLM-17 and MOLM-18

The MOLM-17 and MOLM-18 cell lines were established from a Japanese patient diagnosed with acute myeloid leukemia with monocytic differentiation, corresponding to AML M5, that developed in the setting of advanced myelodysplastic syndrome. Both cell lines originate from the same patient and therefore share a common clonal background, providing a paired in vitro system for studying leukemic transformation and disease progression associated with MDS. Their derivation from overt AML places them biologically beyond primary MDS, and they should be regarded as models of secondary AML arising from antecedent myelodysplasia rather than as bona fide MDS cell lines [30].

Cytogenetic characterization of MOLM-17 and MOLM-18 revealed complex chromosomal abnormalities that are typical of advanced myeloid malignancies and leukemic transformation. Phenotypically, both cell lines display features of monocytic differentiation, consistent with their classification as AML M5. These properties distinguish them from MDS-derived cell lines that retain dysplastic differentiation and cytokine dependence, and instead align them with proliferative leukemic states characterized by blast expansion and relative independence from microenvironmental growth signals [30].

From an experimental standpoint, MOLM-17 and MOLM-18 exhibit robust proliferative capacity and are readily amenable to in vitro culture, making them suitable for large-scale experimental approaches. Their growth characteristics facilitate high-throughput drug screening, functional genomic studies, and signaling pathway analyses that are technically challenging to perform in slow-growing, cytokine-dependent MDS cell lines. Accordingly, these models have been widely used in preclinical testing to evaluate the efficacy of candidate therapeutic agents and to investigate mechanisms of drug response and resistance in advanced myeloid disease [30].

Despite their practical advantages, MOLM-17 and MOLM-18 do not recapitulate key biological hallmarks of primary MDS, including ineffective hematopoiesis, multilineage dysplasia, and strong dependence on extrinsic microenvironmental cues. Their use is therefore most appropriate for studies focused on clonal evolution, leukemic transformation, and secondary AML biology rather than early MDS pathogenesis. Careful consideration of these limitations is essential to avoid overinterpretation of findings when extrapolating results to primary MDS, particularly lower-risk disease states.

### 3.2. Comparative Suitability of MDS and MDS-Related Cell Line Models

The MDS and MDS-related cell lines described in this review differ substantially in their biological characteristics, disease-stage representativeness, and experimental applicability. From a practical standpoint, the selection of an appropriate in vitro model should be guided by the specific biological or translational question under investigation. Cell lines such as MDS92 and its derivative MDS-L retain defining features of dysplastic and ineffective hematopoiesis together with strong cytokine dependence and therefore represent the most faithful available systems for modeling early or preleukemic MDS, particularly high-risk and epigenetically driven disease states. These models are especially informative for studies focused on chromatin regulation and resistance to hypomethylating agents that closely mirror clinically relevant MDS biology.

In contrast, M-TAT and TER-3 preserve measurable differentiation capacity and multilineage features and are best suited for mechanistic investigations of cytokine responsiveness, lineage commitment, and differentiation defects associated with dysplastic hematopoiesis. Their reliance on defined growth factor stimulation makes them particularly useful for dissecting microenvironment-dependent signaling pathways that are difficult to capture in more proliferative models.

Cell lines derived after leukemic transformation, including SKM-1, SKK-1, and MOLM-17 and MOLM-18, originate from secondary AML or advanced disease stages and are characterized by increased proliferative capacity and reduced dependence on extrinsic growth signals. These properties limit their suitability as models of primary or lower-risk MDS but make them valuable systems for studying clonal evolution, leukemic progression, and signaling pathways associated with blast expansion. Accordingly, these models are most appropriately applied in investigations of MDS-to-AML transition, therapeutic resistance, and large-scale pharmacological or functional genomic screening, provided that their inability to recapitulate early-stage MDS biology is explicitly acknowledged.

## 4. Conclusions

MDS represents a significant public health issue, contributing to premature mortality. To date, there has been limited progress in the development of effective therapeutic strategies for MDS.

Research utilizing cell lines derived from MDS patients may enable the identification and targeting of two major mechanisms underlying treatment resistance in MDS: epigenetic dysregulation and clonal heterogeneity. Effective treatment of MDS and AML has the potential to improve patient prognosis and quality of life, while also reducing healthcare-associated costs.

Although MDS comprises a genetically and clinically heterogeneous group of hematopoietic disorders, established MDS-derived cell lines provide a reproducible, scalable, and cost-effective platform for studying disease mechanisms. These models recapitulate many of the key genetic and epigenetic alterations found in patient samples, supporting research into specific MDS subtypes, driver mutations, and the pathogenesis of leukemic transformation. They have facilitated investigations into defective hematopoietic differentiation, apoptosis, and clonal evolution and have been widely applied in high-throughput drug screening.

In this context, careful alignment of the experimental model with the research objective is essential. Studies focused on epigenetic dysregulation and resistance to hypomethylating agents are best supported by bona fide MDS-derived models such as MDS92 and MDS-L, which retain disease-relevant genetic and functional features. Investigations of differentiation impairment and cytokine-dependent signaling are most appropriately conducted using M-TAT or TER-3. Conversely, high-throughput drug screening, signaling pathway interrogation, and studies of leukemic transformation are better served by secondary AML-derived models, including SKM-1 and MOLM-17 and MOLM-18, provided that their limitations in modeling primary MDS biology are explicitly acknowledged.

At the same time, it is essential to distinguish between cell lines that authentically model MDS-associated ineffective hematopoiesis and those derived from secondary AML, which primarily reflect leukemic transformation rather than primary disease biology. Although AML-derived models are valuable for investigating disease progression and therapeutic resistance, their biological properties differ fundamentally from those of MDS, particularly lower-risk subtypes. Clear acknowledgment of these distinctions is critical for appropriate experimental design and for accurate interpretation of preclinical findings. Finally, the MDS cell line model’s utility is limited by the lack of appropriate controls, a gap that may be addressed using primary bone marrow cells or immortalized progenitor models such as hTERT-transduced hematopoietic stem cells.

## Figures and Tables

**Table 1:** Summary characteristics of cell lines used in MDS research.

Cell Line	Clinical Origin (Previous Phase)	Markers	Key Mutations & Cytogenetics	Growth Requirements & Phenotype	Authentication/Availability	Experimental Applications	Ref.
MDS92	MDS-EB1 (originated from RARS)	CD34+, CD41+	Complex karyotype; 5q-, 7q-, 12p-, 13q-; Mutation in *N-ras*	Highly IL-3 dependent; blastic and myeloid morphology	No/Original authors	Modeling preleukemic states and DNMT inhibitor resistance	[22,23]
MDS-L	Subclone of MDS92 (MDS phase)	CD34+, CD117+, CD11b+, CD38+, CD33+,	Complex karyotype; mutations in *CEBPA*, *NRAS*, *TET2*, *TP53* gene disruption in *RB1*, *ROH*	IL-3 dependent (MDS-LGF requires less than MDS-L-2007)	No/Original authors	Indolent high-risk MDS phenotype modeling; apoptosis studies (WFA, rigosertib)	[22,24,25,26]
TER-3	MDS/RAEB (originated from RA → RAEB → RAEB-T)	CD15+, CD19+, CD61+ CD11b-, CD13-, CD14-, CD33-, CD34-	Multilineage phenotype:	Dependent on GM-CSF and IL-3	Yes as MDS/No	Studying G-CSF-mediated differentiation and multilineage transformation	[22,27]
M-TAT	MDS/RAEB (at relapse)	No data	Erythroid and megakaryocytic differentiation potential	Dependent on GM-CSF and IL-3	No/No	Analyzing microenvironmental growth signals and differentiation regulation	[22]
SKK-1	sAML (Terminal) (Previous: RAEB → RAEB-T)	CD11b+, CD13+, CD33+, CD45+, CD64+, CD117+, CD3-, CD4-, CD5-, CD34-,	Trisomy 8; mutation in *BCOR*, *U2AF1*	Autonomous growth	No/Original authors	Studying MDS-to-AML transformation and splicing factor mutations	[22,28]
SKM-1	sAML/Monoblastic (Previous: RAEB-T)	CD4+, CD11b+ CD13+, CD33+, CD45+, CD64+, CD117+, CD2-, CD3-, CD5-, CD7-, CD8-, CD34-,	Monosomy 7, mutation in *ASXL1*, *BCORL1*, *EZH2*, *SF1*, *STAG2*, *TET2*, *TP53*, *WT1*	Autonomous; monocytic differentiation potential	Yes/Yes	Investigating drug resistance, gene silencing (SPAG6), and epigenetic drug screening	[22,28,29]
MOLM-17 MOLM-18	AML M5a (from advanced RAEB-T)	CD4+, CD11a+, CD13+, Cd33+, CD56+, CD117+,	Complex chromosomal aberrations	Autonomous growth; monocyte differentiation	Yes as AML/Original authors	Functional genomics and screening for MDS transformation to AML	[22,30]
PC-MDS	N/A (Initially t-MDS)	CD3−, CD14−, CD15+, CD19−, CD33+, CD71+, CD235a+	BCR-ABL1+	Fast-growing, autonomous mononuclear cells	Misidentified (actually K562)/Yes as K562	Historical context only; not recommended for MDS-specific biology	[22,31]

## Data Availability

No new data were created or analyzed in this study. Data sharing is not applicable to this article.
